# Feature‐Based Patterns Associated With Japan NBI Expert Team Type 1 Classification of Serrated Colorectal Lesions: A Post Hoc Decision Tree Analysis

**DOI:** 10.1002/deo2.70428

**Published:** 2026-09-12

**Authors:** Taku Sakamoto, Hiroyuki Takamaru, Daizen Hirata, Iwatate Mineo, Yasushi Sano, Hideki Ishikawa, Saito Yutaka, the Japan NBI Expert Team (JNET)

**Affiliations:** ^1^ Department of Gastroenterology Institute of Medicine University of Tsukuba Tsukuba Japan; ^2^ Endoscopy Division National Cancer Center Hospital Tokyo Japan; ^3^ Gastrointestinal Center Sano Hospital Kobe Japan; ^4^ Department of Molecular‐Targeting Prevention Kyoto Prefectural University of Medicine Kyoto Japan

**Keywords:** colonic polyps, colonoscopy, decision trees, narrow band imaging, serrated lesions

## Abstract

**Objectives:**

Japan NBI Expert Team (JNET) Type 1 classification is used for optical characterization of colorectal lesions with magnifying NBI, but reported Type 1‐related findings may differ between readers when assessing serrated lesions. This small‐scale post hoc reader‐based study explored reported feature‐classification patterns associated with JNET Type 1 selection among serrated colorectal lesions and compared them between expert and non‐expert endoscopists.

**Methods:**

We analyzed de‐identified data from two web‐based image‐interpretation studies using the same colorectal lesion image dataset. Thirteen histologically confirmed serrated lesions (11 sessile serrated lesions [SSLs] and two hyperplastic polyps [HPs]) assessed by 27 expert and 49 non‐expert Japanese endoscopists were included. Simplified JNET responses were dichotomized as Type 1 versus non‐Type 1. Classification and regression tree analysis was used to visualize associations between this outcome and predefined features, including Type 1‐supportive findings and an anti‐Type 1 composite variable. Variability was assessed using entropy.

**Results:**

In the expert and non‐expert core models, the root splits were anti‐Type 1 findings and regular dark or white spots, respectively. These root splits were stable in leave‐one‐reader‐out analyses, but the non‐expert root split changed to anti‐Type 1 findings after excluding unassessable responses. Median entropy was lower among experts than non‐experts (0.229 vs. 0.954).

**Conclusions:**

This exploratory post hoc decision tree analysis suggested relatively stable expert‐associated feature‐classification patterns, whereas non‐expert patterns were influenced by the treatment of unassessable responses. These findings reflect exploratory associations in Type 1 assignment, not cognitive decision sequences, SSL–HP differentiation, or a validated diagnostic algorithm.

## 1 Introduction

Sessile serrated lesions (SSLs) are important precursors in the serrated neoplasia pathway and key targets for colorectal cancer prevention [1, 2]. Because SSLs are often flat, mucus‐covered, and indistinct from the surrounding mucosa, they are difficult to detect and characterize during routine colonoscopy [3]. Accurate optical characterization is directly linked to appropriate clinical management [4, 5], and missed or misdiagnosed SSLs have been implicated in interval colorectal cancers [6, 7]. Although SSLs have characteristic magnifying narrow‐band imaging (NBI) features, their recognition remains inconsistent, particularly among non‐expert endoscopists [8, 9].

The Japan NBI Expert Team (JNET) classification was developed as a simplified diagnostic framework for colorectal lesions using magnifying NBI [10]. JNET Type 1 is typically associated with non‐neoplastic lesions, including hyperplastic polyps [HPs] and SSLs, and previous studies have shown that JNET classification is useful for differentiating neoplastic from non‐neoplastic lesions [11, 12]. However, JNET Type 1 was not designed to distinguish SSLs from HPs. Therefore, when focusing on serrated lesions, it is important to clarify reported feature‐classification patterns associated with Type 1 assignment, rather than to interpret JNET Type 1 as an SSL‐specific diagnostic category.

The objective of this study was not to assess diagnostic accuracy or SSL–HP differentiation, but to explore reported endoscopic feature‐classification patterns associated with JNET Type 1 selection among serrated lesions. Using classification and regression tree (CART) analysis [13], we aimed to visualize hierarchical associations between predefined endoscopic findings and final JNET Type 1 versus non‐Type 1 classifications, and to compare these feature‐classification patterns between expert and non‐expert endoscopists.

## 2 Methods

### 2.1 Study Design and Data Source

This study was a small‐scale post hoc reader‐based secondary analysis of two previously conducted web‐based image‐interpretation studies that evaluated the application of the JNET classification using magnifying NBI for colorectal lesions [14, 15]. The expert dataset was derived from the study by Sakamoto et al. [14], which included experienced Japanese endoscopists, whereas the non‐expert dataset was derived from the Japanese non‐expert cohort of the international multicenter study by Saito et al. [15]. Both original studies used an identical dataset of 150 histopathologically confirmed colorectal lesions and a standardized web‐based assessment platform. For the present analysis, we extracted 13 histologically confirmed serrated lesions, including 11 SSLs and two HPs. These data yielded 351 expert and 637 non‐expert reader‐lesion responses, for a total of 988 reader‐lesion responses.

### 2.2 Participants and Reading Procedure

A total of 27 expert Japanese endoscopists and 49 non‐expert Japanese endoscopists were included in our post hoc analysis. The non‐expert sample size reflected all available completed responses for the first reading session in the source dataset used for this secondary analysis.

All participants independently reviewed the endoscopic images online and completed a structured questionnaire that included the JNET classification (Q19) and predefined endoscopic findings related to vascular and surface patterns (Table 1). For the sessions analyzed in the present study, each lesion was evaluated using sequential still images, consisting of white‐light imaging, non‐magnified NBI, and magnified NBI. Final JNET classification and feature assessments were recorded after this stepwise review. Clinical contextual information outside the standardized image set was not incorporated into this reader‐based analysis.

**TABLE 1:** Web‐based questionnaire items for endoscopic feature assessment.

Category	Item no.	Endoscopic finding/Question	Clinical significance/JNET Classification
Vascular pattern	Q1	Evaluability of vessel pattern	—
	Q2	Invisible vessels	JNET Type 1
	Q3	Regular vessel caliber	JNET Type 1
	Q4	Regular vessel distribution	JNET Type 2A
	Q5	Variable vessel caliber	JNET Type 2B
	Q6	Irregular vessel distribution	JNET Type 2A
	Q7	Loose vessel areas	JNET Type 3
	Q8	Interruption of thick vessels	JNET Type 3
Surface pattern	Q9	Evaluability of surface pattern	—
	Q10	Regular dark or white spots	JNET Type 1
	Q11	Similar to surrounding normal mucosa	JNET Type 1
	Q12	Regular (tubular, branched, or papillary)	JNET Type 2A
	Q13	Irregular or obscure pattern	JNET Type 2B
	Q14	Amorphous areas	JNET Type 3
Additional findings	Q15	Scattered vessels	Deep submucosal invasion
	Q16	Thick, linear, or meandering vessels	Deep submucosal invasion
	Q17	Demarcated area	Deep submucosal invasion
Diagnostic assessment	Q18–20	JNET diagnosis, confidence, and histology	Overall assessment
SSL‐specific features	Q21	VMV or DBV (vessel finding)	SSL‐specific finding^*^
	Q22	Irregular dark spot (surface finding)	SSL‐specific finding^*^

^*^ Items Q21 and Q22 were included only in the evaluation for Japanese expert endoscopists.

Expert assessments were derived from the second reading session of the expert study, whereas non‐expert assessments were derived from the first reading session of the non‐expert study. These sessions were selected because both used the same sequential image‐presentation protocol and therefore provided comparable visual information flow between reader groups. The first expert session was not used because it did not provide the same sequential image flow, and the second non‐expert session was not used because it was performed after e‐learning and therefore did not represent baseline interpretation. Only responses corresponding to the 13 serrated lesions were extracted for further analysis.

### 2.3 Variables and Outcome Definition

Simplified JNET classification responses were recorded as Q19_2: 0 = unassessable; 1 = Type 1; 2 = Type 2A; 3 = Type 2B; and 4 = Type 3. For the decision tree analyses, the outcome variable was defined as the selection of JNET Type 1 versus non‐Type 1. Responses with Q19_2 = 1 were classified as Type 1 selected, whereas responses with Q19_2 = 0, 2, 3, or 4 were classified as non‐Type 1. This primary outcome definition reflected Type 1 selection versus Type 1 non‐selection; therefore, unassessable responses were included in the non‐Type 1 category in the primary analysis because Type 1 was not selected.

Questionnaire items capturing endoscopic findings were used to derive clinically defined binary and composite explanatory variables for decision tree analyses. Type 1‐supportive findings included invisible vessels (Q2), regular vessel caliber (Q3), regular dark or white spots (Q10), and similarity to the surrounding normal mucosa (Q11). The anti‐Type 1 composite variable was defined as the presence of at least one finding inconsistent with JNET Type 1 classification, including findings suggestive of Type 2A, Type 2B, Type 3, or deep submucosal invasion (Q4–Q8 and Q12–Q17). SSL‐specific secondary findings (Q21–Q22) were assessed only in the expert study design and were not presented to non‐expert readers.

### 2.4 Assessment of Inter‐reader Variability

Inter‐reader variability in the JNET classification was assessed for each lesion using Shannon entropy. To characterize variability among interpretable responses, entropy was calculated using only valid responses (Q19_2 = 1–4), excluding unassessable responses (Q19_2 = 0). For each lesion, the modal category (mode) and mode rate, defined as the proportion of valid responses selecting the modal category, were calculated. The number of unassessable responses (Q19_2 = 0) was descriptively reported for each lesion and reader group (Table 2).

**TABLE 2:** Inter‐reader variability in simplified Japan NBI Expert Team (JNET) classification (Q19_2) for each serrated lesion assessed by expert and non‐expert endoscopists.

Case no.	Mode (Expert)	Entropy (Expert)	Mode rate (Expert)	Unassessable *n* (Expert)	Mode (Non‐expert)	Entropy (Non‐expert)	Mode rate (Non‐expert)	Unassessable *n* (Non‐expert)
1	Type 1	0.000	1.000	0	Type 1	0.925	0.778	4
2	Type 1	0.229	0.963	0	Type 1	1.121	0.543	3
3	Type 1	0.951	0.630	0	Type 2A	0.997	0.532	2
4	Type 1	0.000	1.000	1	Type 1	0.599	0.854	1
5	Type 1	0.000	1.000	0	Type 1	1.140	0.692	10
6	Type 1	0.000	1.000	0	Type 1	1.211	0.737	7
7	Type 1	0.000	1.000	0	Type 2B	1.965	0.667	0
8	Type 1	0.235	0.962	1	Type 1	0.415	0.933	4
9	Type 2A	0.999	0.519	0	Type 2A	0.736	0.851	2
10	Type 1	0.229	0.963	0	Type 1	0.787	0.860	6
11	Type 1	0.000	1.000	0	Type 1	1.639	0.581	6
12	Type 1	0.000	1.000	0	Type 1	0.966	0.783	3
13	Type 2A	0.229	0.963	0	Type 2A	0.954	0.625	1

The entropy and mode rate were calculated using only the valid responses (Q19_2 = 1–4). Responses marked as unassessable (Q19_2 = 0) were excluded from these calculations; the number of unassessable responses is shown for reference.

### 2.5 Decision Tree Analysis for Visualization of Feature‐Classification Patterns

Decision tree analyses were performed using the CART approach implemented in Python with scikit‐learn to visualize hierarchical associations between reported endoscopic features and final JNET Type 1 versus non‐Type 1 classifications. To enhance interpretability and avoid overly complex trees based on highly correlated individual questionnaire items, explanatory variables were clinically grouped and dichotomized before model construction. The core model included binary variables representing Type 1‐supportive findings, including invisible vessels, regular vessel caliber, regular dark or white spots, and similarity to the surrounding normal mucosa, together with a composite variable indicating the presence of any anti‐Type 1 finding. The anti‐Type 1 composite variable was defined as the presence of at least one finding inconsistent with JNET Type 1 classification, including findings suggestive of Type 2A, Type 2B, Type 3, or deep submucosal invasion.

Analyses were conducted separately for expert and non‐expert groups using identical model parameters to enable comparison of feature‐classification structures. For experts, two models were constructed: (1) a core model using JNET‐related supportive and anti‐Type 1 findings and (2) an extended model that additionally incorporated SSL‐specific secondary findings. In the extended model, SSL‐specific secondary findings were summarized as a composite variable indicating the presence of VMV/DBV or irregular dark spots (Q21 or Q22). For non‐experts, only the core model was constructed because Q21–Q22 were not assessed in the non‐expert study protocol and were not treated as absent findings. Analyses were conducted using Python version 3.12.13 with scikit‐learn version 1.6.1. The DecisionTreeClassifier algorithm was applied using the Gini impurity criterion, with a minimum leaf size of five reader‐lesion responses and a random state of 0. The primary purpose of these analyses was to visualize exploratory feature‐classification patterns rather than to optimize predictive performance. The resulting trees were interpreted as hierarchical associations between reported features and final classifications, not as evidence of the temporal or cognitive sequence by which endoscopists evaluated individual features.

### 2.6 Sensitivity Analyses of CART‐Derived Root Splits

To assess root‐split stability, we performed leave‐one‐lesion‐out and leave‐one‐reader‐out sensitivity analyses by iteratively excluding one lesion or all responses from one reader, respectively, and refitting each tree using the original model specification. Parameter sensitivity analyses used alternative combinations of minimum leaf size and maximum tree depth. To assess the influence of unassessable responses, trees were refitted after excluding responses with Q19_2 = 0. These analyses evaluated exploratory root‐split stability, not predictive validity.

## 3 Results

### 3.1 Inter‐reader Variability in Simplified JNET Classification for Serrated Lesions

The inter‐reader variability in the simplified JNET classification (Q19_2) for the 13 serrated lesions was assessed using Shannon entropy calculated from valid responses only (Q19_2 = 1–4), excluding unassessable responses (Q19_2 = 0). The median entropy across lesions was 0.229 (interquartile range [IQR], 0.000–0.235) in the expert group and 0.954 (IQR, 0.736–1.121) in the non‐expert group (Table 2).

Unassessable responses were more frequently identified among non‐experts than among experts. The median number of unassessable responses per lesion was four (range: 1–10) in the non‐expert group, compared with 0 (range: 0–1) in the expert group (Table 2).

**FIGURE 1:**
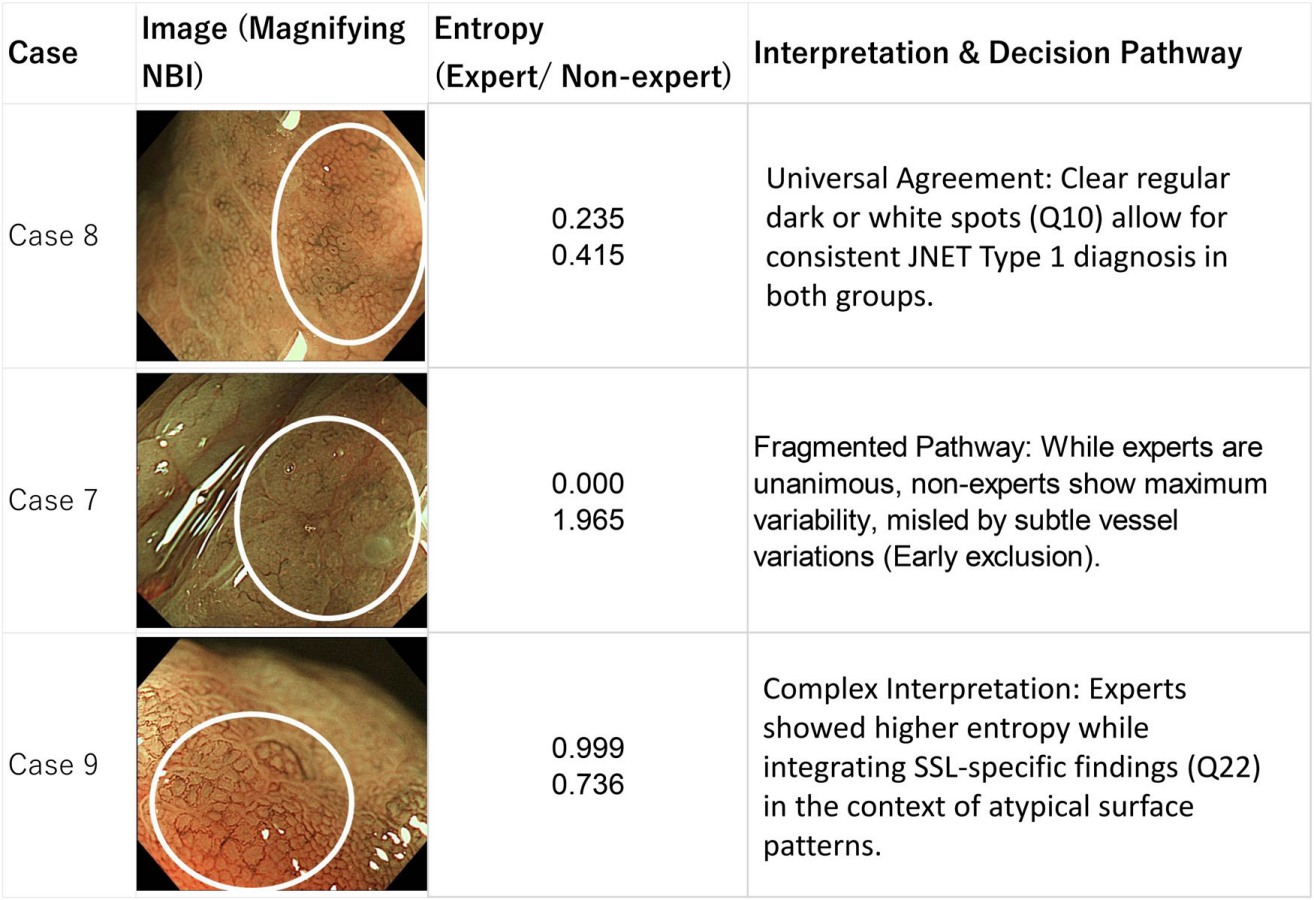
Representative magnifying narrow‐band imaging (NBI) images and inter‐reader variability data. Representative cases illustrate different patterns of inter‐reader variability based on the quantitative data in Table 2. (A) Case 8: A Japan NBI Expert Team (JNET) Type 1 lesion with low entropy in both groups, in which regular dark or white spots (Q10) were frequently reported. (B) Case 7: A case with marked variability among non‐experts. Experts showed unanimous Type 1 assignment (entropy 0.000), whereas non‐experts showed high variability (entropy 1.965). (C) Case 9: A case with higher entropy among experts, in which sessile serrated lesion (SSL)‐specific secondary findings, such as irregular dark spots (Q22), were reported in the context of atypical surface patterns.

To complement the entropy‐based assessment of variability, modal agreement was summarized using the mode rate. The median mode rate across lesions was 0.963 (IQR: 0.963–1.000) in the expert group and 0.737 (IQR: 0.581–0.851) in the non‐expert group (Table 2). Representative cases illustrating different patterns of inter‐reader variability are shown in Figure 1.

### 3.2 Visualization of Feature‐Classification Patterns via Decision Tree Analysis

**FIGURE 2:**
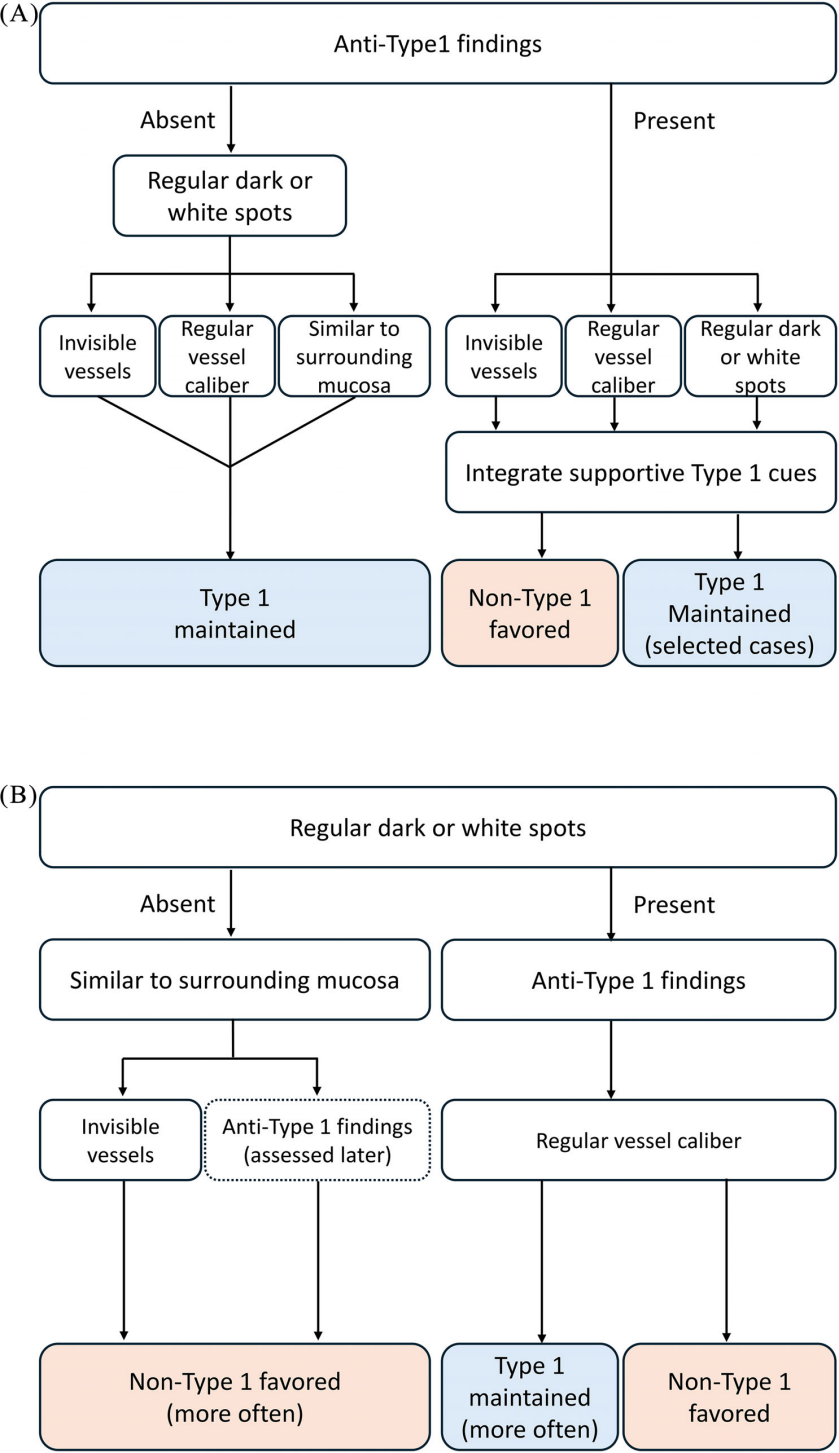
Decision tree analysis of feature‐classification patterns based on core Japan NBI Expert Team (JNET)‐related features. (A) Decision tree illustrating feature‐classification patterns associated with JNET Type 1 selection among expert endoscopists. In the expert core model, anti‐Type 1 findings formed the root split. Downstream branches included supportive Type 1 features, such as regular dark or white spots, invisible vessels, regular vessel caliber, and similarity to the surrounding normal mucosa. (B) Decision tree illustrating feature‐classification patterns among non‐expert endoscopists. In the non‐expert core model, regular dark or white spots formed the root split, whereas anti‐Type 1 findings appeared in downstream branches. For clarity, simplified trees are shown; full structures are provided in Figures S1 and S2.

Decision tree analysis was performed to visualize hierarchical associations between reported endoscopic features and JNET Type 1 selection for serrated lesions. In the expert core model, anti‐Type 1 findings formed the root split associated with JNET Type 1 selection. When anti‐Type 1 findings were absent, terminal nodes were predominantly classified as Type 1. When anti‐Type 1 findings were present, downstream splits included regular dark or white spots, invisible vessels, regular vessel caliber, and similarity to the surrounding normal mucosa (Figure 2A).

In the non‐expert core model, regular dark or white spots formed the root split associated with JNET Type 1 selection. Anti‐Type 1 findings appeared in downstream branches, together with other Type 1‐supportive features. Compared with the expert core model, the non‐expert tree showed a different feature‐classification structure, with the root split based on a Type 1‐supportive surface finding rather than the anti‐Type 1 composite variable (Figure 2B).

**FIGURE 3:**
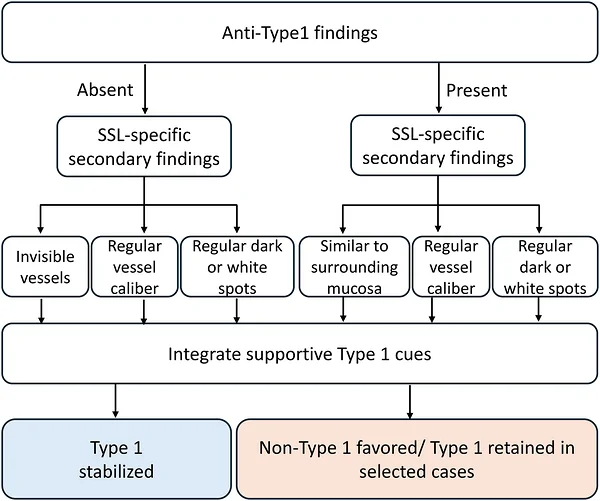
Expert extended model incorporating sessile serrated lesion (SSL)‐specific secondary findings. Decision tree analysis among expert endoscopists incorporating SSL‐specific secondary findings in addition to core Japan NBI Expert Team (JNET)‐related features. In the full dataset, anti‐Type 1 findings formed the root split. SSL‐specific secondary findings appeared in downstream branches and were interpreted as exploratory contextual variables associated with Type 1 assignment among serrated lesions. These findings are not components of the original JNET classification and should not be interpreted as replacing or redefining the core JNET‐related framework. A simplified tree is shown; the full structure is provided in Figure S3.

In the expert extended model incorporating SSL‐specific secondary findings, anti‐Type 1 findings remained the root split in the full dataset. SSL‐specific secondary findings appeared in downstream branches, particularly in subsets of responses in which anti‐Type 1 findings were present (Figure 3).

### 3.3 Sensitivity Analyses

In sensitivity analyses, the original root splits of the expert core and non‐expert core models were retained across all leave‐one‐lesion‐out iterations, all leave‐one‐reader‐out iterations, and all alternative tree specifications. Specifically, anti‐Type 1 findings remained the root split in the expert core model, whereas regular dark or white spots remained the root split in the non‐expert core model. In the expert extended model, anti‐Type 1 findings remained the root split in 9 of 13 leave‐one‐lesion‐out iterations, 21 of 27 leave‐one‐reader‐out iterations, and all alternative tree specifications; SSL‐specific secondary findings formed the root split in the remaining 4 lesion‐level and 6 reader‐level iterations. After excluding unassessable responses, the root splits were retained in the expert core and expert extended models. In contrast, the non‐expert core model root split changed from regular dark or white spots to anti‐Type 1 findings. Details are shown in Table S1.

## 4 Discussion

This small‐scale post hoc reader‐based study used CART as an exploratory visualization tool to examine feature‐classification patterns associated with JNET Type 1 selection for serrated colorectal lesions. The resulting trees summarize hierarchical associations between reported features and final Type 1 versus non‐Type 1 classifications, not the temporal or cognitive sequence of assessment.

In the primary analysis, the expert core model was rooted in anti‐Type 1 findings, whereas the non‐expert core model was rooted in regular dark or white spots. This suggests that Type 1 assignment was more closely associated with the absence of Type 1‐inconsistent findings among experts and with a supportive surface finding among non‐experts. However, after excluding unassessable responses, the non‐expert root split changed to anti‐Type 1 findings, indicating that the apparent between‐group difference in the primary analysis was partly influenced by the treatment of unassessable responses.

The clinical interpretation of these findings requires caution. JNET Type 1 classification was developed primarily within a framework for distinguishing non‐neoplastic from neoplastic colorectal lesions and estimating the likelihood of deeper invasion in neoplastic lesions; it was not designed to differentiate SSLs from HPs. Therefore, the present analysis does not propose a new diagnostic method for SSL–HP differentiation or resection decision‐making. Its relevance lies instead in identifying feature‐classification patterns that may help explain why non‐expert readers show greater variability when assigning JNET Type 1 to serrated lesions.

Among the 13 lesions included in this analysis, 11 were histologically confirmed as SSLs and only two as HPs, precluding meaningful subgroup analyses comparing SSLs and HPs. This imbalance reflects the available histologically confirmed serrated lesions in the original magnifying NBI image dataset; small distal HPs are often not resected and may therefore be underrepresented in datasets requiring histopathological confirmation. Accordingly, the findings should be interpreted as feature‐classification patterns among serrated lesions enriched for SSLs, rather than as evidence applicable equally to SSLs and HPs.

Another consideration is that neoplastic lesions potentially misclassified as Type 1 were excluded. In the source datasets, such misclassification was uncommon: Sakamoto et al. reported that only 1.1% of neoplastic lesions were misclassified as Type 1 [14], and Saito et al. reported a similarly low misclassification rate of 1.4% for high‐grade or malignant lesions [15]. These findings provide some reassurance that the focused analysis of serrated lesions was unlikely to overlook a major competing pattern of neoplastic lesions being assigned to Type 1 under magnifying NBI. However, this design also means that the present study cannot evaluate how readers distinguish serrated lesions from neoplastic lesions in routine practice.

A strength of this study is that all participating endoscopists evaluated the same 13 lesions using a standardized web‐based protocol. Although the number of lesions was limited, this reader‐based design yielded 988 reader‐lesion responses and allowed direct comparison of reader‐dependent variability and feature‐classification patterns under constant lesion characteristics. Thus, the present study should be interpreted as an exploratory reader‐based analysis rather than as a lesion‐level diagnostic validation study. However, SSL‐specific secondary findings were evaluated only by expert endoscopists in the original study design. Therefore, analyses incorporating these findings were restricted to the expert group and should be interpreted as exploratory within‐expert analyses rather than as comparisons with non‐expert interpretation.

The role of SSL‐specific secondary findings should also be interpreted carefully. VMV/DBV and irregular dark spots have been reported as SSL‐specific endoscopic features [16, 17], but they are not components of the original JNET classification and should not be interpreted as redefining JNET Type 1. Reviews have emphasized integrating clinicopathological features, image‐enhanced findings, and management context rather than relying on a single classification label [18, 19]. In the expert‐only extended model, these findings appeared as contextual variables associated with Type 1 assignment among serrated lesions. Because sensitivity analyses showed that the root split of the expert extended model was less stable than those of the core models, SSL‐specific secondary findings should be regarded as exploratory contextual variables rather than components of a validated JNET‐based decision algorithm.

From an educational perspective, these findings should be viewed as hypothesis‐generating. Future training modules could emphasize not only recognition of Type 1‐supportive findings, such as regular dark or white spots, but also how findings inconsistent with Type 1 may affect final classification. Whether such training improves diagnostic accuracy, confidence, or inter‐reader consistency requires prospective validation.

This study had several limitations. First, only 13 histologically confirmed serrated lesions, including 2 HPs, were analyzed, limiting lesion‐level generalizability. Second, the reader–lesion responses were clustered by reader and lesion. Third, CART does not capture the temporal or cognitive sequence of endoscopic assessment. Fourth, the non‐expert core model was sensitive to the treatment of unassessable responses, and the reasons for Q19_2 = 0 responses could not be determined. Fifth, all evaluations used still images from a single institution without clinical context or real‐time endoscopic manipulation. Finally, the study did not evaluate SSL–HP differentiation or resection decision‐making, and its educational implications require prospective validation.

In conclusion, this exploratory post hoc decision tree analysis suggested that expert‐associated feature‐classification patterns for JNET Type 1 assignment were relatively stable, with anti‐Type 1 findings consistently forming the root split in the expert core model. In the primary non‐expert model, regular dark or white spots formed the root split, but this pattern was influenced by the treatment of unassessable responses. These findings should be interpreted as exploratory associations rather than evidence of cognitive decision sequences, SSL–HP differentiation, or validated diagnostic algorithms. Future studies are needed to determine whether these patterns can inform educational interventions and improve diagnostic performance or consistency in clinical practice.

## 5 Declaration of Generative Artificial Intelligence Use

OpenAI ChatGPT was used to assist with preparing analysis code and improving the clarity of selected text. All decision tree analyses, including sensitivity analyses, were subsequently rerun and verified by the authors using Python/scikit‐learn. The authors reviewed all outputs, figures, and interpretations and take full responsibility for the content of the manuscript. No artificial intelligence tool was used to generate, alter, or manipulate original research data.

## Author Contributions

**Taku Sakamoto**: conceptualization, methodology, formal analysis, Writing – original draft, and visualization. **Hiroyuki Takamaru**: data curation, investigation, and writing – review & editing. **Daizen Hirata**: investigation and data curation. **Mineo Iwatate**: investigation and methodology. **Yasushi Sano**: supervision and validation. **Hideki Ishikawa**: formal analysis and methodology. **Yutaka Saito**: project administration, supervision, and writing – review & editing. All authors have read and approved the final version of the manuscript.

## Funding

This work was supported in part by the National Cancer Center Research and Development Funds [grant numbers 29‐A‐13, 2020‐A‐12, and 2023‐A‐15].

## Ethics Statement

This study was approved by the Institutional Review Board (IRB) of the National Cancer Center Hospital (Approval No: 2018–082) and registered in the University Hospital Medical Information Network (UMIN) Clinical Trials Registry (UMIN ID: 000039307).

## Consent

The IRB waived the requirement for informed consent for participation in the study, as this was a retrospective analysis of data obtained during standard clinical care. All patients had provided written informed consent for the colonoscopy and endoscopic treatment.

## Conflicts of Interest

The authors declare no conflicts of interest.

## Supporting information




**FILE S1** deo270428‐sup‐0001‐FigureS1.pdf

**FILE S2** deo270428‐sup‐0002‐FigureS2.pdf

**FILE S3** deo270428‐sup‐0003‐FigureS2.pdf

**FIGURE S1** Full decision tree of the expert core model based on core JNET‐related features. The first and second pages show the absent and present branches of the root split, respectively, with the root node repeated on each page to indicate the branch location within the same tree.
**FIGURE S2** Full decision tree of the non‐expert core model based on core JNET‐related features. The first and second pages show the absent and present branches of the root split, respectively, with the root node repeated on each page to indicate the branch location within the same tree.
**FIGURE S3** Full decision tree of the expert extended model incorporating SSL‐specific secondary findings. The first and second pages show the absent and present branches of the root split, respectively, with the root node repeated on each page to indicate the branch location within the same tree.
In Figures S1–S3, “Absent” and “Present” indicate absence and presence of the split feature, respectively. Node labels show the split feature or terminal classification, the number of reader‐lesion responses (n), and the percentage of responses classified as JNET Type 1. Anti‐Type 1 findings indicate the composite variable defined by the presence of any finding inconsistent with JNET Type 1 classification (Q4–Q8 and Q12–Q17). Regular dark/white spots, invisible vessels, regular vessel caliber, and similar to normal mucosa correspond to Type 1‐supportive findings Q10, Q2, Q3, and Q11, respectively. SSL‐specific findings indicate the presence of VMV/DBV or irregular dark spots (Q21 or Q22).
**FILE S4** deo270428‐sup‐0004‐TableS1.docx
**TABLE S1** Sensitivity analyses of CART‐derived root splits.
Supporting Information

## Data Availability

The data supporting the findings of this study are not publicly available because they were derived from previously conducted image‐interpretation studies. Additional information may be available from the corresponding author upon reasonable request and with appropriate approval.
