# Supplementary material for: Feature‐Based Patterns Associated With Japan NBI Expert Team Type 1 Classification of Serrated Colorectal Lesions: A Post Hoc Decision Tree Analysis
Source: DEN Open. 2026 Sep 12;7(1):e70428. doi: 10.1002/deo2.70428 (PMC13570418; doi:10.1002/deo2.70428)
Supplement: Supplementary file 1 [file DEO2-7-e70428-s001.pdf]

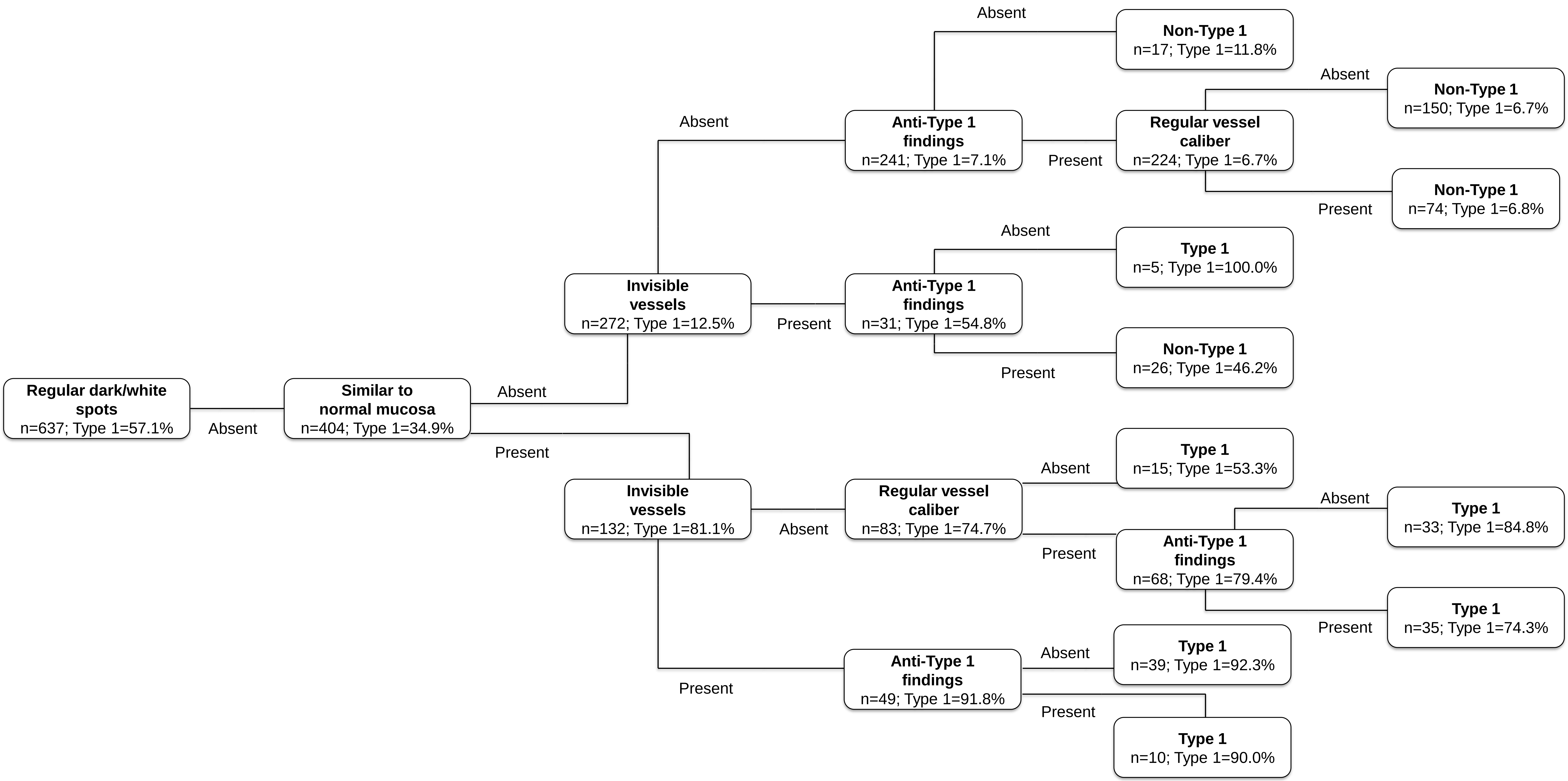
Regular dark/white spots
n=637; Type 1=57.1%
Absent
Similar to normal mucosa
n=404; Type 1=34.9%
Absent
Present
Invisible vessels
n=272; Type 1=12.5%
Absent
Present
Anti-Type 1 findings
n=241; Type 1=7.1%
Absent
Present
Non-Type 1
n=17; Type 1=11.8%
Regular vessel caliber
n=224; Type 1=6.7%
Absent
Present
Non-Type 1
n=150; Type 1=6.7%
Non-Type 1
n=74; Type 1=6.8%
Anti-Type 1 findings
n=31; Type 1=54.8%
Absent
Present
Type 1
n=5; Type 1=100.0%
Non-Type 1
n=26; Type 1=46.2%
Invisible vessels
n=132; Type 1=81.1%
Absent
Present
Regular vessel caliber
n=83; Type 1=74.7%
Absent
Present
Type 1
n=15; Type 1=53.3%
Anti-Type 1 findings
n=68; Type 1=79.4%
Absent
Present
Type 1
n=33; Type 1=84.8%
Type 1
n=35; Type 1=74.3%
Anti-Type 1 findings
n=49; Type 1=91.8%
Absent
Present
Type 1
n=39; Type 1=92.3%
Type 1
n=10; Type 1=90.0%

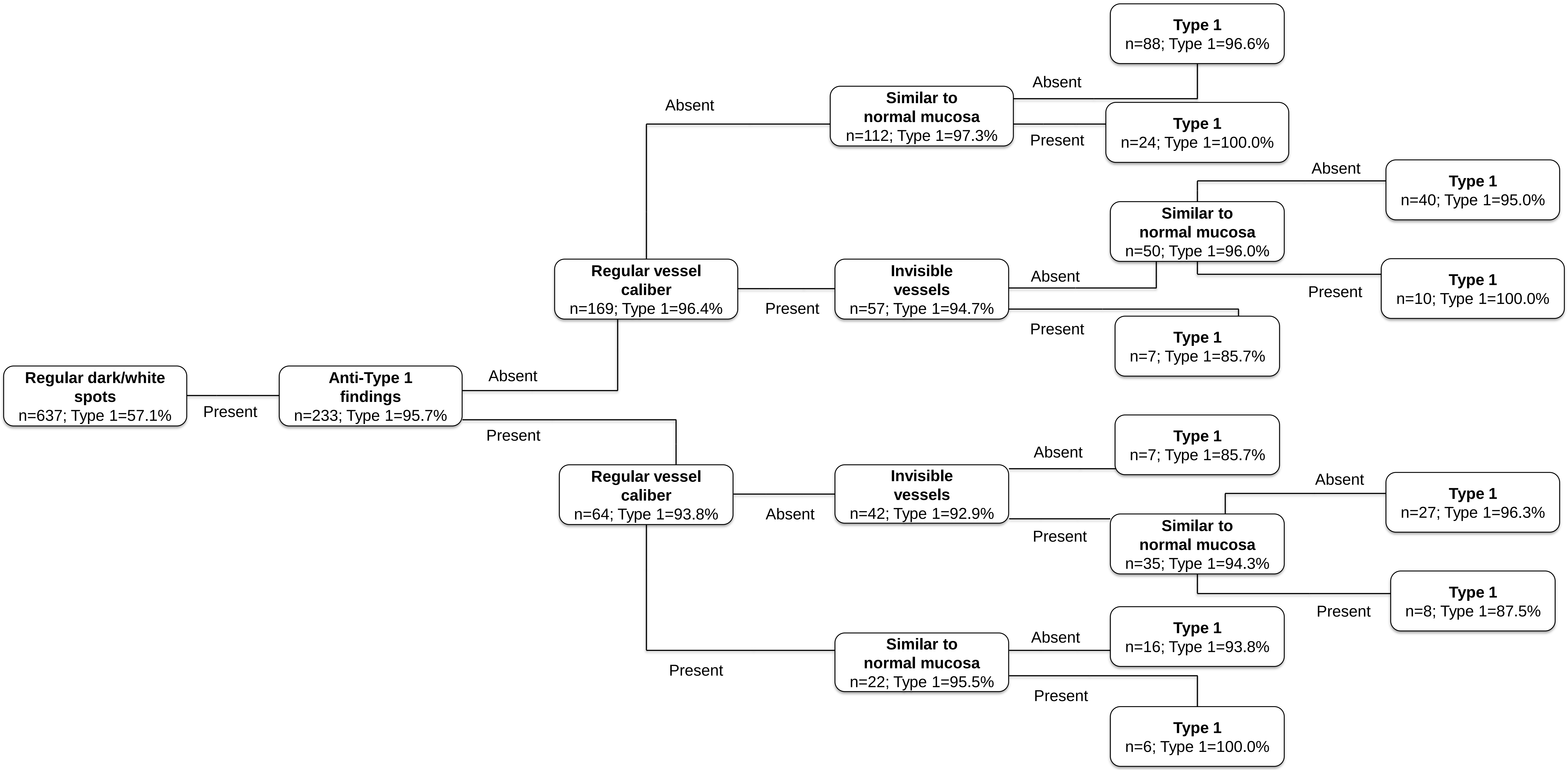

Regular dark/white spots
n=637; Type 1=57.1%
Present
Anti-Type 1 findings
n=233; Type 1=95.7%
Absent
Regular vessel caliber
n=169; Type 1=96.4%
Absent
Similar to normal mucosa
n=112; Type 1=97.3%
Absent
Type 1
n=88; Type 1=96.6%
Present
Type 1
n=24; Type 1=100.0%
Present
Invisible vessels
n=57; Type 1=94.7%
Absent
Similar to normal mucosa
n=50; Type 1=96.0%
Absent
Type 1
n=40; Type 1=95.0%
Present
Type 1
n=10; Type 1=100.0%
Present
Type 1
n=7; Type 1=85.7%
Present
Regular vessel caliber
n=64; Type 1=93.8%
Absent
Invisible vessels
n=42; Type 1=92.9%
Absent
Type 1
n=7; Type 1=85.7%
Present
Similar to normal mucosa
n=35; Type 1=94.3%
Absent
Type 1
n=27; Type 1=96.3%
Present
Type 1
n=8; Type 1=87.5%
Present
Similar to normal mucosa
n=22; Type 1=95.5%
Absent
Type 1
n=16; Type 1=93.8%
Present
Type 1
n=6; Type 1=100.0%
