# Supplementary material for: Feature‐Based Patterns Associated With Japan NBI Expert Team Type 1 Classification of Serrated Colorectal Lesions: A Post Hoc Decision Tree Analysis
Source: DEN Open. 2026 Sep 12;7(1):e70428. doi: 10.1002/deo2.70428 (PMC13570418; doi:10.1002/deo2.70428)
Supplement: Supplementary file 2 [file DEO2-7-e70428-s002.pdf]

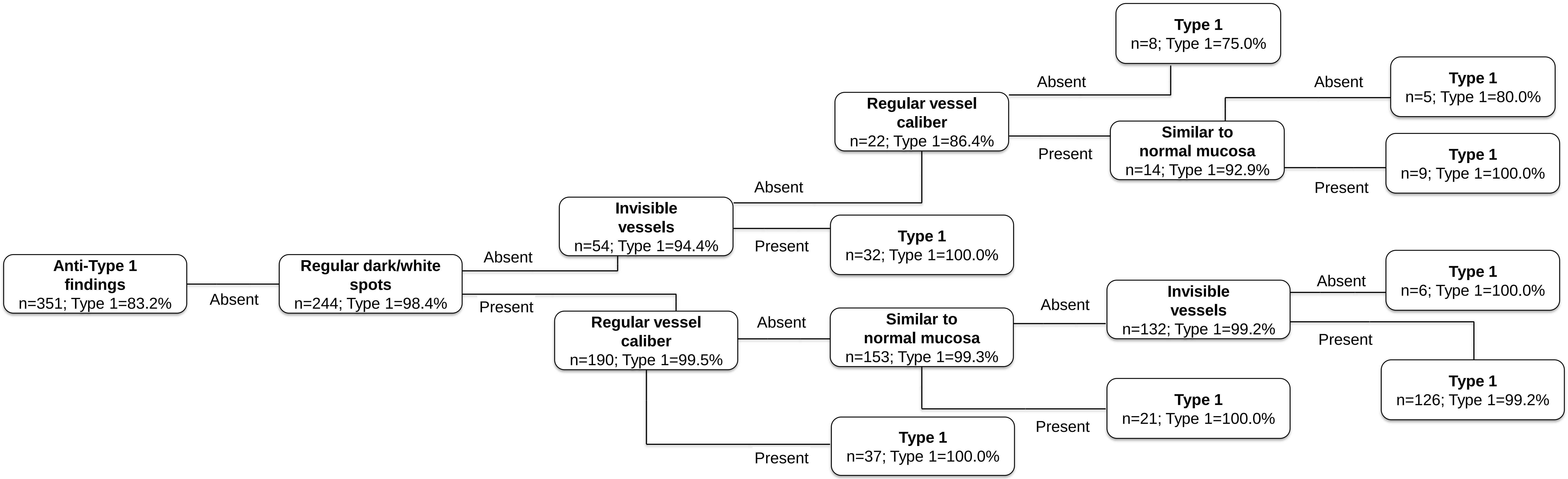

Anti-Type 1 findings
n=351; Type 1=83.2%
Absent
Regular dark/white spots
n=244; Type 1=98.4%
Absent
Invisible vessels
n=54; Type 1=94.4%
Absent
Regular vessel caliber
n=22; Type 1=86.4%
Absent
Type 1
n=8; Type 1=75.0%
Present
Similar to normal mucosa
n=14; Type 1=92.9%
Absent
Type 1
n=5; Type 1=80.0%
Present
Type 1
n=9; Type 1=100.0%
Present
Type 1
n=32; Type 1=100.0%
Present
Regular vessel caliber
n=190; Type 1=99.5%
Absent
Similar to normal mucosa
n=153; Type 1=99.3%
Absent
Invisible vessels
n=132; Type 1=99.2%
Absent
Type 1
n=6; Type 1=100.0%
Present
Type 1
n=126; Type 1=99.2%
Present
Type 1
n=21; Type 1=100.0%
Present
Type 1
n=37; Type 1=100.0%

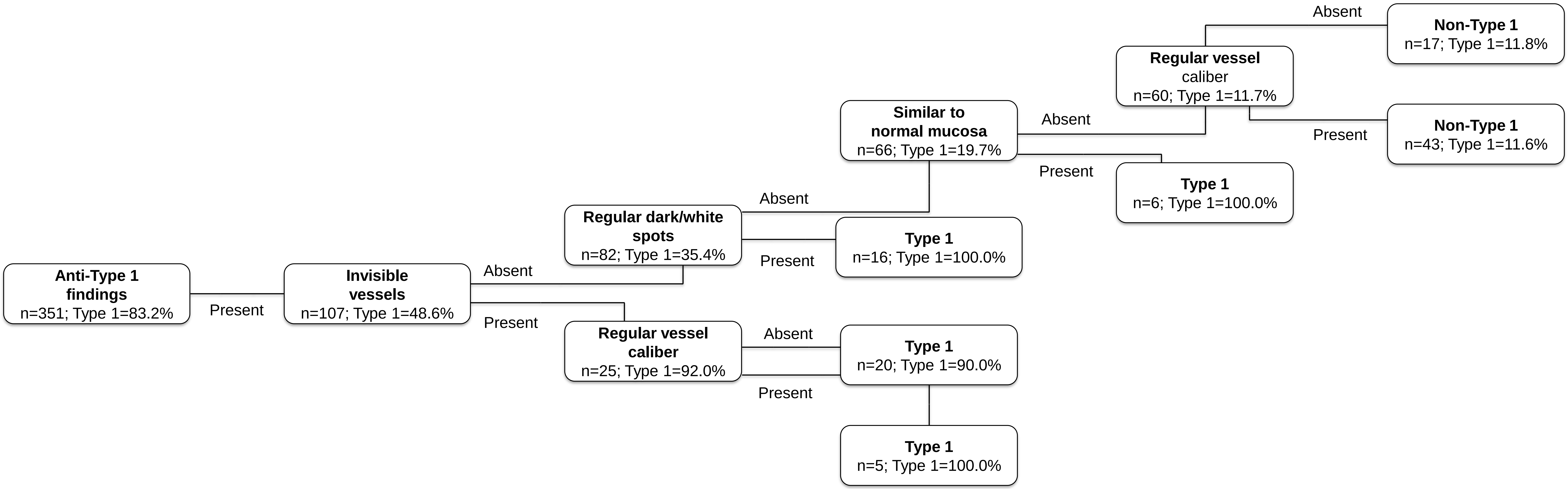
Anti-Type 1 findings
n=351; Type 1=83.2%
Present
Invisible vessels
n=107; Type 1=48.6%
Absent
Regular dark/white spots
n=82; Type 1=35.4%
Absent
Similar to normal mucosa
n=66; Type 1=19.7%
Absent
Regular vessel caliber
n=60; Type 1=11.7%
Absent
Non-Type 1
n=17; Type 1=11.8%
Present
Non-Type 1
n=43; Type 1=11.6%
Present
Type 1
n=6; Type 1=100.0%
Present
Type 1
n=16; Type 1=100.0%
Present
Regular vessel caliber
n=25; Type 1=92.0%
Absent
Type 1
n=20; Type 1=90.0%
Present
Type 1
n=5; Type 1=100.0%
