# Supplementary material for: Feature‐Based Patterns Associated With Japan NBI Expert Team Type 1 Classification of Serrated Colorectal Lesions: A Post Hoc Decision Tree Analysis
Source: DEN Open. 2026 Sep 12;7(1):e70428. doi: 10.1002/deo2.70428 (PMC13570418; doi:10.1002/deo2.70428)
Supplement: Supplementary file 3 [file DEO2-7-e70428-s003.pdf]

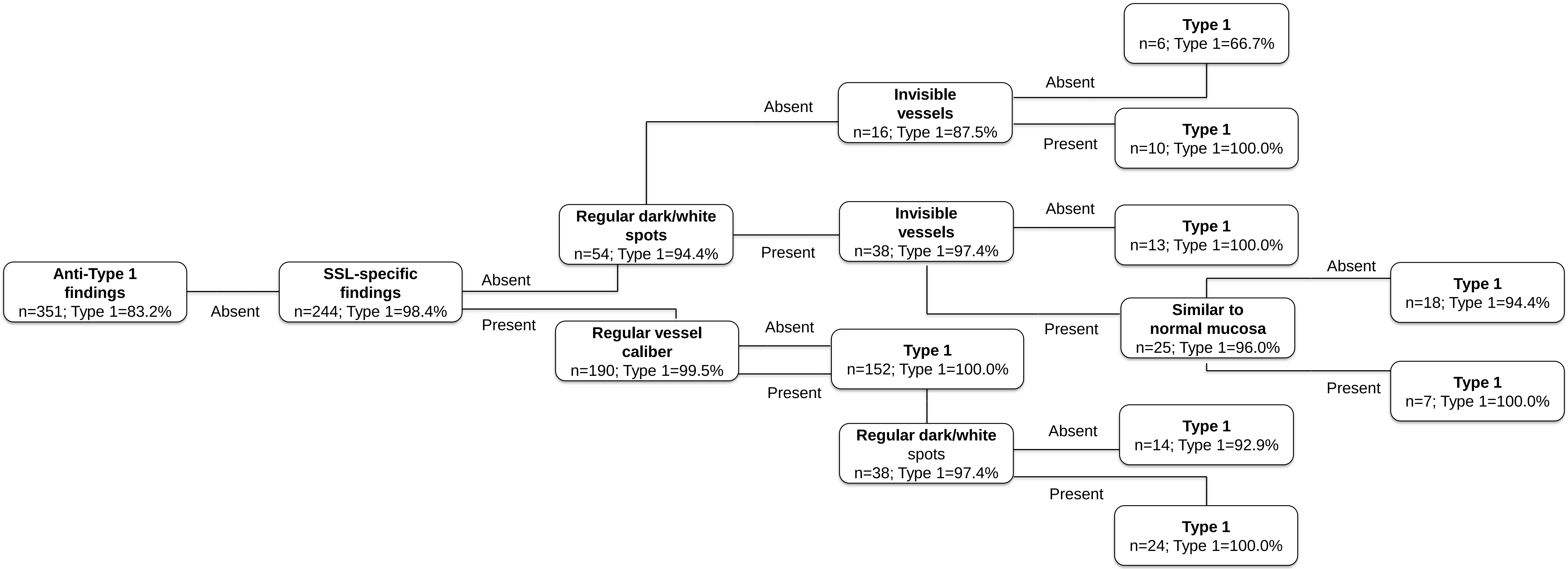

Anti-Type 1 findings
n=351; Type 1=83.2%
Absent
SSL-specific findings
n=244; Type 1=98.4%
Absent
Present
Regular dark/white spots
n=54; Type 1=94.4%
Absent
Present
Invisible vessels
n=16; Type 1=87.5%
Absent
Type 1
n=6; Type 1=66.7%
Present
Type 1
n=10; Type 1=100.0%
Invisible vessels
n=38; Type 1=97.4%
Absent
Type 1
n=13; Type 1=100.0%
Present
Similar to normal mucosa
n=25; Type 1=96.0%
Absent
Type 1
n=18; Type 1=94.4%
Present
Type 1
n=7; Type 1=100.0%
Regular vessel caliber
n=190; Type 1=99.5%
Absent
Present
Type 1
n=152; Type 1=100.0%
Regular dark/white spots
n=38; Type 1=97.4%
Absent
Type 1
n=14; Type 1=92.9%
Present
Type 1
n=24; Type 1=100.0%

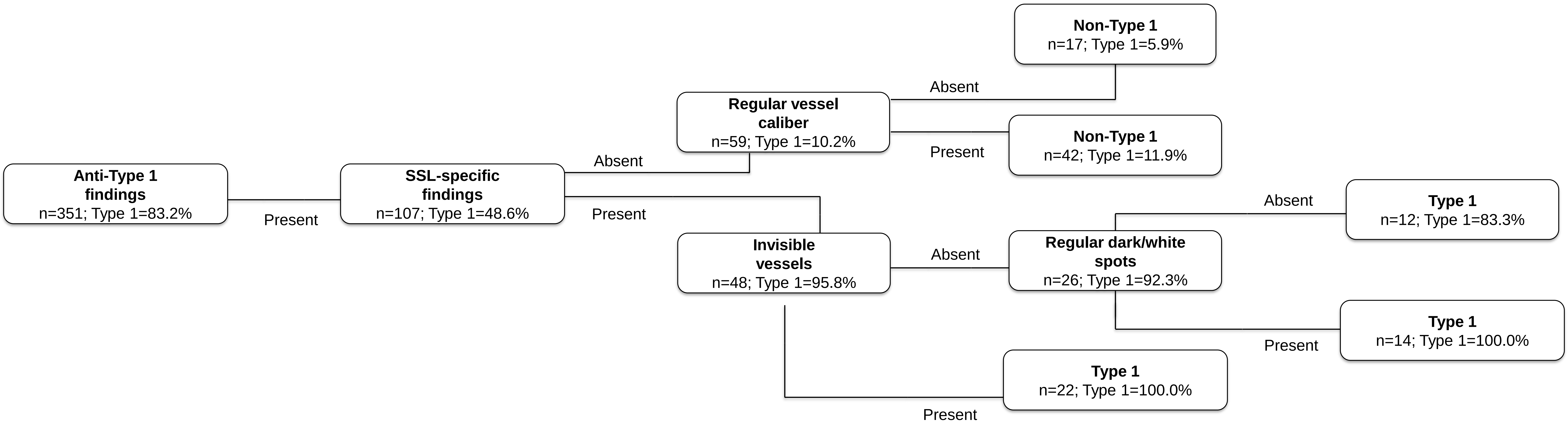
Anti-Type 1
findings
n=351; Type 1=83.2%
Present
SSL-specific
findings
n=107; Type 1=48.6%
Absent
Present
Regular vessel
caliber
n=59; Type 1=10.2%
Absent
Present
Non-Type 1
n=17; Type 1=5.9%
Non-Type 1
n=42; Type 1=11.9%
Invisible
vessels
n=48; Type 1=95.8%
Absent
Present
Regular dark/white
spots
n=26; Type 1=92.3%
Absent
Present
Type 1
n=12; Type 1=83.3%
Type 1
n=14; Type 1=100.0%
Type 1
n=22; Type 1=100.0%
