# Supplementary material for: Feature‐Based Patterns Associated With Japan NBI Expert Team Type 1 Classification of Serrated Colorectal Lesions: A Post Hoc Decision Tree Analysis
Source: DEN Open. 2026 Sep 12;7(1):e70428. doi: 10.1002/deo2.70428 (PMC13570418; doi:10.1002/deo2.70428)
Supplement: Supplementary file 4 [file DEO2-7-e70428-s004.docx]

**Supplementary Table S1. Sensitivity analyses of CART-derived root splits**

| Model | Reader-lesion responses | Feature set | Original root split | Leave-one-lesion-out analysis | Leave-one-reader-out analysis | Parameter sensitivity analysis | Analysis excluding unassessable responses | Interpretation |
| --- | --- | --- | --- | --- | --- | --- | --- | --- |
| Expert core model | 351 | Core JNET-related features | Anti-Type 1 findings | 13/13 retained | 27/27 retained | 12/12 retained | Retained (Anti-Type 1 findings) | Stable across lesion-level, reader-level, parameter, and unassessable-response sensitivity analyses. |
| Non-expert core model | 637 | Core JNET-related features | Regular dark or white spots | 13/13 retained | 49/49 retained | 12/12 retained | Changed to Anti-Type 1 findings | Stable across lesion-level, reader-level, and parameter sensitivity analyses, but sensitive to the treatment of unassessable responses. |
| Expert extended model | 351 | Core JNET-related features plus SSL-specific secondary findings | Anti-Type 1 findings | 9/13 retained; changed to SSL-specific secondary findings in 4/13 iterations | 21/27 retained; changed to SSL-specific secondary findings in 6/27 iterations | 12/12 retained | Retained (Anti-Type 1 findings) | Generally preserved, but showed partial variability when SSL-specific secondary findings were included. |

**Abbreviations:** CART, classification and regression tree; JNET, Japan NBI Expert Team; SSL, sessile serrated lesion.

**Footnotes:**

- Values indicate the number of iterations or model specifications in which the original root split was retained. Alternative root splits are described only when observed.

- Leave-one-lesion-out analysis was performed by iteratively excluding one of the 13 serrated lesions and refitting the decision tree using the original model specification: Gini criterion, minimum leaf size of 5, and random state of 0.

- Leave-one-reader-out analysis was performed by iteratively excluding all responses from one reader and refitting the decision tree using the original model specification.

- Parameter sensitivity analysis was performed by refitting the trees under 12 alternative model specifications using minimum leaf sizes of 5, 10, 15, and 20 crossed with maximum tree depths of 2, 3, and 4.

- Unassessable responses were defined as responses with Q19_2 = 0. The analysis excluding unassessable responses was performed after removing these responses and refitting each decision tree.

- Core JNET-related features included invisible vessels, regular vessel caliber, regular dark or white spots, similarity to surrounding mucosa, and the composite variable of anti-Type 1 findings.

- Anti-Type 1 findings were defined as the presence of any feature inconsistent with JNET Type 1 classification.

- SSL-specific secondary findings were defined as the presence of VMV/DBV or irregular dark spots.

- In non-experts, SSL-specific secondary findings were not included because Q21-Q22 were not assessed in the original non-expert protocol and were therefore not treated as absent findings.

- These sensitivity analyses were intended to evaluate the stability of CART-derived root splits as exploratory feature-classification patterns, not to validate a predictive diagnostic algorithm or to infer the temporal or cognitive sequence of endoscopic assessment.
